# The cytoskeleton adaptor protein ankyrin-1 is upregulated by p53 following DNA damage and alters cell migration

**DOI:** 10.1038/cddis.2016.91

**Published:** 2016-04-07

**Authors:** A E Hall, W-T Lu, J D Godfrey, A V Antonov, C Paicu, S Moxon, T Dalmay, A Wilczynska, P A J Muller, M Bushell

**Affiliations:** 1 Medical Research Council (MRC), Toxicology Unit, Leicester, UK; 2 The Genome Analysis Centre, Norwich, UK; 3 School of Computing Sciences, University of East Anglia, Norwich, UK; 4 School of Biological Sciences, University of East Anglia, Norwich, UK

## Abstract

The integrity of the genome is maintained by a host of surveillance and repair mechanisms that are pivotal for cellular function. The tumour suppressor protein p53 is a major component of the DNA damage response pathway and plays a vital role in the maintenance of cell-cycle checkpoints. Here we show that a microRNA, miR-486, and its host gene *ankyrin-1* (*ANK1*) are induced by p53 following DNA damage. Strikingly, the cytoskeleton adaptor protein ankyrin-1 was induced over 80-fold following DNA damage. *ANK1* is upregulated in response to a variety of DNA damage agents in a range of cell types. We demonstrate that miR-486-5p is involved in controlling G1/S transition following DNA damage, whereas the induction of the ankyrin-1 protein alters the structure of the actin cytoskeleton and sustains limited cell migration during DNA damage. Importantly, we found that higher *ANK1* expression correlates with decreased survival in cancer patients. Thus, these observations highlight *ANK1* as an important effector downstream of the p53 pathway.

Cells respond to DNA damage by orchestrating a series of events, either resulting in cell-cycle arrest and DNA repair, or elimination of the damaged cell. DNA double-strand breaks (DSB) are one of the most toxic types of DNA damage experienced by cells.^1^ A complex network of mechanisms has evolved to detect and repair DSBs. DNA repair is achieved either via non-homologous end-joining or in a more accurate manner by homologous recombination.^2^ Failure of either of these mechanisms triggers apoptosis.^3^

The DNA damage response pathway (DDR) involves a cascade of events with multiple effector components,^3, 4, 5, 6, 7^ key to which is the tumour suppressor protein p53.^8^ DNA damage leads to stabilisation of p53 resulting from the degradation of its ubiquitin ligase, MDM2.^9^ This induces the transcription of genes whose products trigger cell-cycle arrest, DNA repair or apoptosis.^7^ More recently, p53 has been shown to regulate certain microRNAs (miRNA) by facilitating their transcription or modulating the activity of the miRNA biogenesis machinery.^10^

MiRNAs are ~22 nt RNA molecules which base pair with target mRNAs resulting in translation inhibition and destabilisation of the bound mRNA.^11^ These small RNAs are involved in a range of biological processes and regulate more than half of all protein-coding genes.^11^ For example, the transcriptional activation of the miR-34 family by p53 following DNA damage^12^ results in the inhibition of key targets, including the transcription factor c-Myc which controls genes involved in cell-cycle progression and cell growth.^13, 14^

In addition to its roles in cell death, p53 has also been implicated in cell motility,^15^ and mutant p53 promotes tumour cell invasion and results in loss of directionality during migration.^16^ Cell migration is a complex process and is controlled by many proteins,^17^ and the specific role of p53 in this mechanism is not yet completely understood.

Here, we initially set out to identify new miRNAs associated with DDR. We found miR-486-5p levels increased ~8-fold following DNA damage, and to our surprise, found the host gene *ANK1* increased ~80-fold. We show that both miR-486 and *ANK1* are regulated by p53 and that miR-486-5p is involved in controlling G1/S transition following DNA damage. On the other hand, ankyrin-1 plays a role in sustaining cell motility through actin cytoskeleton remodelling upon non-apoptotic levels of DNA damage. Importantly, we found that high *ANK1* levels correlate with reduced survival in cancer patients.

## Results

### Identification of miRNAs upregulated following DNA damage

**Figure 1:**
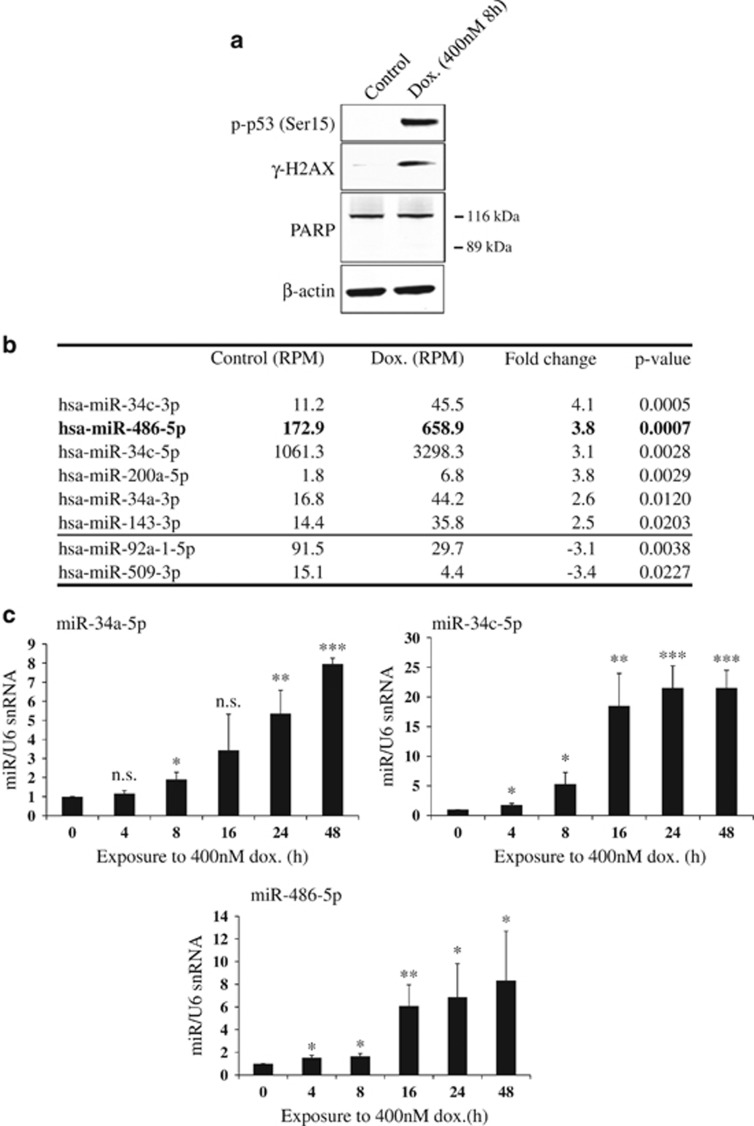
Identification of microRNAs upregulated following DNA damage. (**a**) DNA damage was induced in MCF10A cells with doxorubicin (Dox.) at a concentration of 400 nM for 8 h, and was confirmed by western blots for phospho-p53 (Ser15) and *γ*-H2AX. Absence of cleaved PARP (89 kDa) confirmed cells were not undergoing apoptosis. Representative western blots shown (*n*=3). (**b**) Small RNA deep sequencing (Illumina) was carried out on total RNA from control and DNA damaged MCF10A cells. MicroRNAs with a significant fold-change (*P*<0.05) (*n*=3) are shown, with normalised reads displayed as reads per million (RPM). (**c**) Validation of miR-486-5p upregulation following DNA damage in MCF10A cells that had been exposed to doxorubicin (400 nM) for 0–48 h. MiR-486-5p levels were quantified via qPCR, as well as miR-34 family members as a comparison. Values are mean±S.D. (*t*-test, *n*=3, compared with 0 h control), *P<0.05, **P<0.01 and ***P<0.001; NS, not significant. Corresponding western blots are shown in Figure 2c

To identify miRNAs that change following DNA damage, we treated the non-tumorigenic MCF10A cell line with the DNA topoisomerase II inhibitor doxorubicin to induce DSBs^18^ (Figure 1a) and subjected them to small RNA deep sequencing (Figure 1b). Induction of histone H2A.X phosphorylation (*γ*-H2AX, Ser139) and p53 phosphorylation (Ser15) confirmed the activation of DDR.^4^ The dose and duration of damage were chosen to avoid apoptotic induction, confirmed by the absence of PARP cleavage (Figure 1a and Supplementary Figure S1).

Analysis revealed six miRNAs that showed significant upregulation following damage (*P*<0.05), all of which showed >2-fold change (Figure 1b). Two miRNAs were downregulated ~3-fold (*P*<0.05) (miR-92a-1-5p and miR-509-3p). Among the upregulated miRNAs were members of the miR-34 family, including miR-34c and miR-34a which are known to be induced following DNA damage;^19, 20, 21^ miR-143-3p which has been previously implicated in DDR;^22^ and miR-200a which has a well-known role in cancer metastasis^23^ and DNA damage checkpoint control.^24^ Importantly, we also found that miR-486-5p increased by 3.8-fold following DNA damage and was relatively abundant, suggesting a possible functional role in DDR. MiR-486 is a muscle-enriched miRNA, but relatively little is known about its involvement in DDR.^25^

To validate these results and determine the temporal nature of miR-486-5p induction, we performed a doxorubicin time-course using MCF10A cells. MiR-34a and miR-34c were used as positive controls (Figure 1c). As expected, miR-34a increased 5.4-fold after 24 h of doxorubicin treatment and 8.0-fold after 48 h. MiR-34c increased 21.5-fold after 24 h of doxorubicin treatment and remained high at 48 h. A significant 1.5-fold increase in miR-486-5p was detectable as early as 4 h following doxorubicin exposure, and increased to 8.3-fold after 48 h. We therefore selected miR-486 for further investigation.

### The miR-486 host gene *ANK1* is also induced following DNA damage

**Figure 2:**
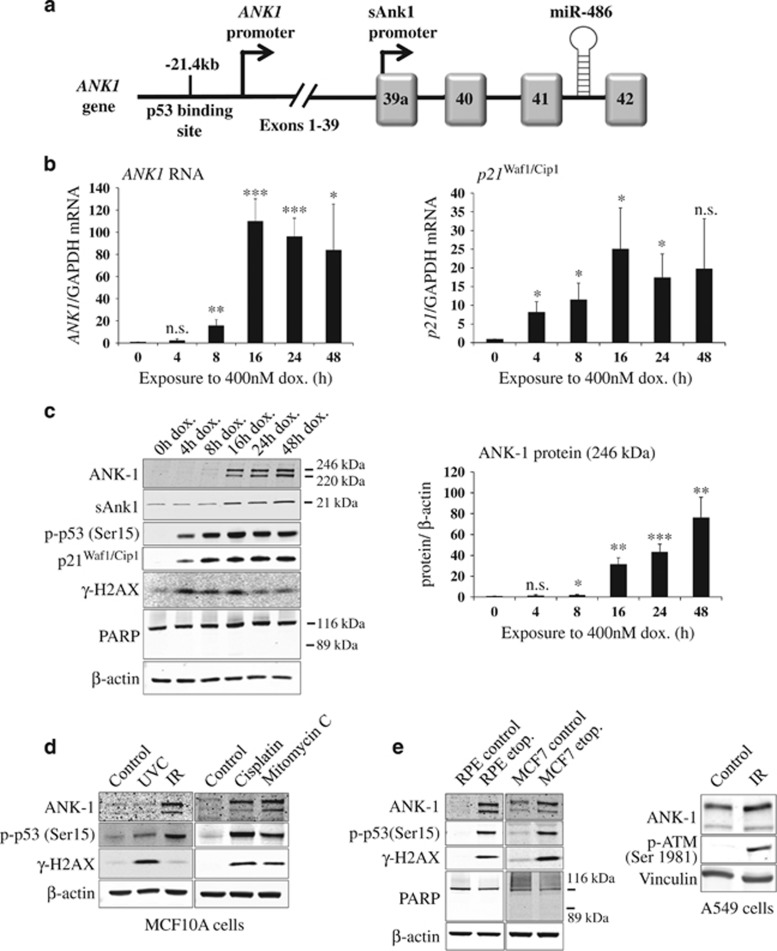
*ANK1* is upregulated following exposure to different inducers of DNA damage and in a variety of cell types. (**a**) Diagram showing the location of miR-486 in relation to the open reading frame of the cytoskeleton adaptor gene *ANK1*, as well as the small ankyrin-1 isoform (sAnk1). MiR-486 is located in the intronic region between the last two exons of the *ANK1* gene. (**b**) MCF10A cells were treated with doxorubicin (dox.) as in Figure 1c, and *ANK1* mRNA levels were quantified by qPCR. The p53-regulated *p21*^(Waf1/ Cip1)^ transcript served as a positive control (*n*=3). (**c**) Representative western analysis of protein samples harvested from cells undergoing DNA damage (*n*=3). The ankyrin-1 protein (ANK1) (246 kDa) was quantified (right panel) and normalised to *β*-actin levels. (**d**) Ankyrin-1 was induced following ionising radiation (IR, 10 Gy) exposure in MCF10A cells but not by ultraviolet light-C (UVC, 100 J/m^2^). Cells were harvested 24 h after IR/UVC exposure. Cisplatin (10 *μ*g/ml) and mitomycin C (300 nM) treatment for 24 h also induced ankyrin-1. Representative western blots are shown (*n*=3). (**e**) Ankyrin-1 was upregulated following 25 *μ*M etoposide (etop.) treatment for 24 h in retinal pigment epithelium (RPE) and MCF7 cells, and in A549 cells following IR (5 Gy) exposure where cells were harvested after 6 h. Representative western blots are shown (*n*=3). For all bar charts, values are mean±S.D. (*t*-test, *n*=3, compared with 0 h control), *P<0.05, **P<0.01 and ***P<0.001; NS, not significant

Approximately half of the 2588 miRNAs in the human genome are intragenic,^26^ and there is often a functional relationship between the miRNA and its host gene.^27^ Intragenic miRNAs can be regulated either by the host gene's promoter or an independent promoter.^28^ MiR-486 is located within the last intron of the cytoskeleton adaptor gene *ANK1* (Figure 2a). Therefore we asked if the primary host gene transcript, *ANK1*, was also induced following DNA damage. It was previously shown that miR-486 is controlled by a p53 promoter located 21.4 kb upstream of *ANK1,*^29^ but to our knowledge no one had previously examined the role of *ANK1* in relation to DNA damage, miR-486 or activation of the p53 pathway.

Strikingly, we found that *ANK1* mRNA was upregulated 16-fold after 8 h of doxorubicin-induced DNA damage (Figure 2b) and 110-fold after 16 h, which was markedly higher than the increase in miR-486-5p expression (Figure 1c). To compare this with a well-known DNA damage-induced transcript, we measured mRNA levels of the p53-regulated gene *p21*^Waf1/Cip1^, which showed a 25-fold increase following 16 h of doxorubicin treatment (Figure 2b). The dramatic induction of *ANK1* mRNA expression levels led us to investigate whether the ankyrin-1 protein was similarly induced. Indeed, we observed that the 246 kDa ankyrin-1 protein was induced ~31-fold after 16 h of doxorubicin treatment (Figure 2c), and ~76-fold after 48 h. Importantly, this was restricted to the canonical ankyrin-1 protein, as the shorter, muscle-enriched isoform of ankyrin-1 (sAnk1) only increased by ~2-fold after 24 h of treatment (Figure 2c).

**Figure 3:**
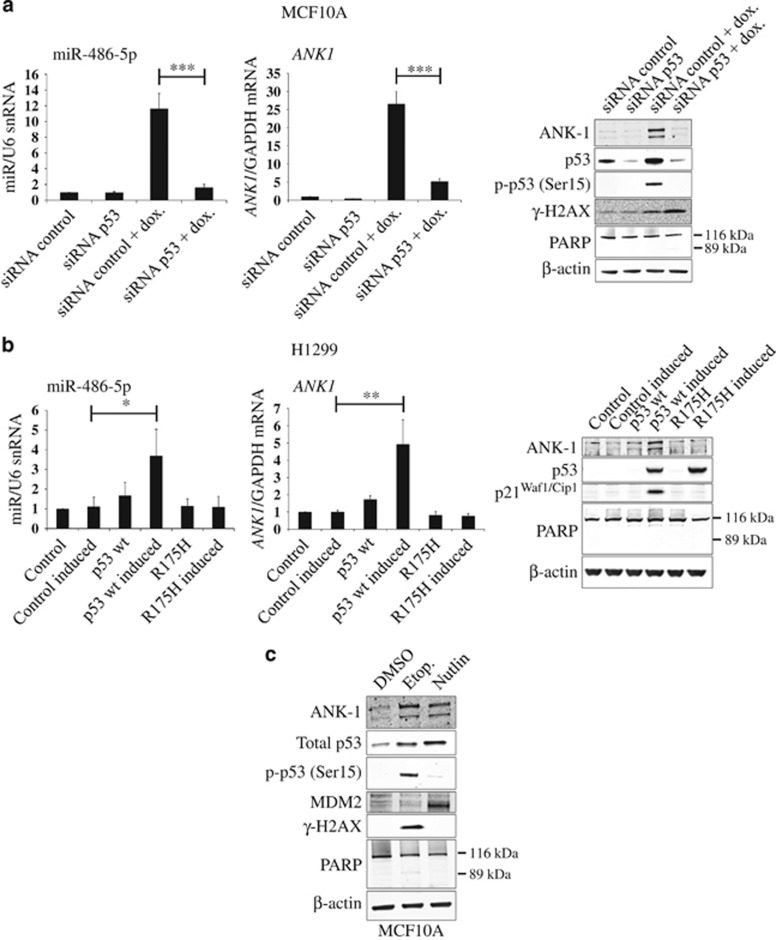
*ANK1* and miR-486 expression are p53 dependent. (**a**) MCF10A cells were subjected to DNA damage using 400 nM doxorubicin (dox.) for 24 h following 48 h siRNA-mediated depletion of p53 or a control siRNA. *ANK1* and miR-486-5p RNA levels were determined by qPCR and representative western blots show ankyrin-1 (ANK1), *γ*-H2AX and phospho-p53 (Ser15) levels following DNA damage and p53 depletion (*n*=3). (**b**) Doxycycline inducible wild-type p53 or mutant p53^R175H^ H1299 cells were either treated (induced) or not treated with doxycycline. Wild type or mutant p53 expression was confirmed via western blot of p53 and p21^(Waf1/ Cip1)^, and *ANK1* and miR-486-5p RNA levels were determined by qPCR (*n*=3). (**c**) Cells were treated with the p53 activator, nutlin. Total p53 and ankyrin-1 levels were analysed by western blot and compared with DNA damage-induced cells treated with the topoisomerase II inhibitor etoposide (etop.) (25 *μ*M) for 24 h. Representative western blots are shown (*n*=3). For bar charts, values are mean±S.D. (*t*-test, *n*=3), *P<0.05, **P<0.01 and ***P<0.001

Next we examined if ankyrin-1 levels increased following exposure to other DNA damage inducers. We found that ionising radiation (IR) exposure induced ankyrin-1 expression in MCF10A cells (Figure 2d), despite low levels of the DNA damage marker *γ*-H2AX. Ankyrin-1 levels were also induced by the DNA crosslinking reagents cisplatin and mitomycin C (Figure 2d), and also by a different DNA topoisomerase II inhibitor, etoposide (Figure 3c). Ultraviolet-C irradiation did not markedly induce p53 phosphorylation or *ANK1* expression, although high levels of *γ*-H2AX were observed (Figure 2d).

To examine if the upregulation of ankyrin-1 was specific to MCF10A cells, we treated another non-tumorigenic cell line, retinal pigment epithelium (RPE) cells, and also breast cancer-derived MCF7 cells with etoposide^18^ (Figure 2e). Both RPE and MCF7 cells showed a marked increase of ankyrin-1 protein levels following DNA damage. Ankyrin-1 was also induced by IR exposure in A549 lung carcinoma cells (Figure 2e).

Taken together, these data demonstrated that *ANK1* was induced in response to various DNA damaging agents in different cell lines, suggesting an important role for *ANK1* in DDR activation.

### *ANK1* and miR-486 expression are p53 dependent

To investigate if the transcription factor p53 was co-regulating both miR-486 and *ANK1* following DNA damage, we depleted p53 with siRNA prior to doxorubicin treatment (Figure 3a). In control cells, DNA damage induced miR-486-5p expression by 11.6-fold and *ANK1* expression by 26.5-fold. However, knockdown of p53 impaired the induction of both miR-486-5p and *ANK1* (Figure 3a).

We then used p53-null cells that harbour either a doxycycline-inducible wild-type p53 or dominant-negative mutant p53^R175H^ construct (Figure 3b).^30^ Upon induction of wild-type p53 expression with doxycycline, a 3.7-fold induction of miR-486-5p and a 4.9-fold increase in *ANK1* mRNA were observed. Importantly, ankyrin-1 was also induced at the protein level (Figure 3b). As expected, expression of mutant p53^R175H^ did not induce p21^Waf1/Cip1^. Likewise, both miR-486-5p and *ANK1* were not induced by mutant p53, demonstrating that only wild-type p53 could induce their expression.

Finally, we asked whether MCF10A cells treated with the p53 activator nutlin^31^ could induce *ANK1* expression independently of DNA damage. In control cells ankyrin-1 was barely detectable (Figure 3c); however, its expression was induced following treatment with etoposide or nutlin. The stabilisation of p53 by nutlin was confirmed by MDM2 and p53 western analysis (Figure 3c).

Taken together, these data strongly suggest that both miR-486 and *ANK1* are induced following DNA damage in a p53-dependent manner.

### miR-486-5p inhibition reverses DNA damage-induced cell-cycle arrest

**Figure 4:**
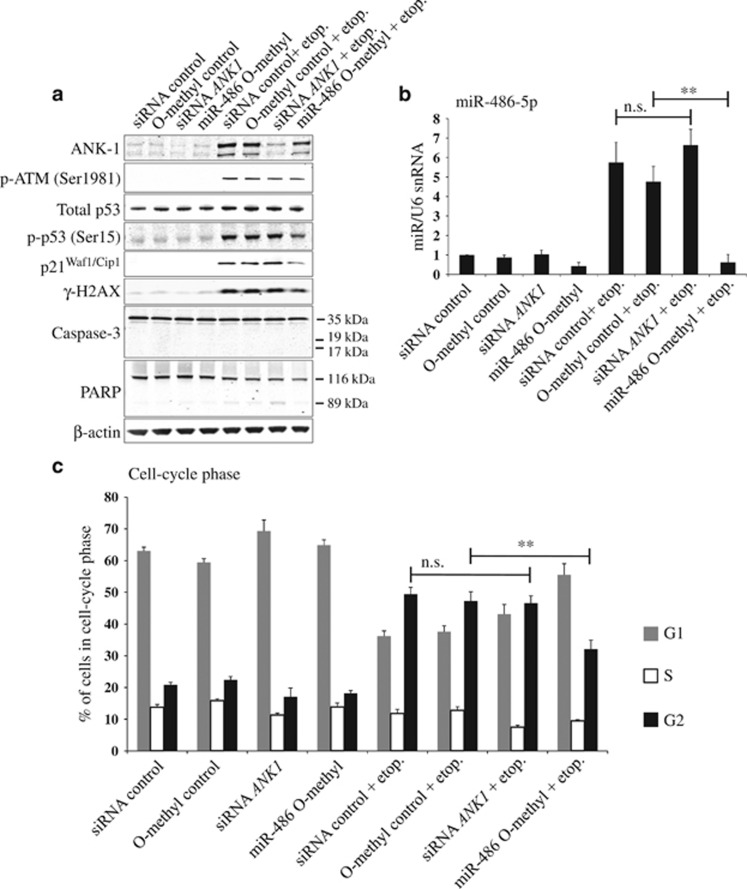
miR-486-5p inhibition partly reverses the DNA damage-induced accumulation of cells in G2. (**a**) Cells were either transfected with a negative control siRNA, an siRNA against *ANK1*, a negative control 2′-*O*-methyl inhibitor or a 2′-*O*-methyl inhibitor against miR-486-5p. After 24 h, DNA damage was induced using 25 *μ*M etoposide (etop.) for 24 h. DNA damage was assessed by p-ATM, p-p53 (Ser15) and *γ*-H2AX while a lack of cleaved caspase-3 and PARP showed that the treatment had not induced apoptosis. Representative western blots are shown (*n*=3). (**b**) miR-486-5p levels were quantified via qPCR (*n*=3). (**c**) Cell-cycle analysis was achieved via propidium iodide staining and subsequent flow cytometry. For all bar charts, values are mean±S.D. (*t*-test, *n*=3), **P<0.01; NS, not significant

Previously it has been reported that miR-486 expression can inhibit cell growth resulting in cell-cycle arrest in G1.^25, 29, 32, 33^ However, these publications did not examine the role of miR-486 following DNA damage in conjunction with the role of the host gene *ANK1* in cell-cycle control. To clarify this, we first set out to establish the individual contributions that increased ankyrin-1 or miR-486-5p levels had on cells following DNA damage. We used siRNA to deplete *ANK1* and anti-miRs to inhibit miR-486-5p (Figures 4a and b). We confirmed that siRNA depletion of *ANK1* resulted in the loss of ankyrin-1 induction following DNA damage (Figure 4a), but did not affect the induction of miR-486-5p (Figure 4b). Likewise, the inhibition of miR-486-5p resulted in ablation of the miRNA induction post-DNA damage (Figure 4b), but did not significantly affect the induction of ankyrin-1 protein (Figure 4a). Now that we could separate the contributions of the mRNA and miRNA components, we examined the individual effects of *ANK1* and miR-486-5p on cell-cycle arrest following DNA damage (Figure 4c). We used etoposide treatment for the fluorescence-activated cell sorting (FACS) experiments because doxorubicin and propidium iodide have overlapping fluorescence emission wavelengths. In control siRNA-transfected cells treated with etoposide, we observed a significant decrease in the G1 population, with a corresponding increase in G2, implying G2/M arrest. Activation of DDR was confirmed by detection of phospho-ATM, phospho-p53 and *γ*-H2A.X (Figure 4a). The depletion of ankyrin-1 following DNA damage (Figure 4a) did not have any effect on the proportion of cells arrested in G2 (Figure 4c). This suggests that DNA damage-induced G2 arrest was *ANK1*-independent. Importantly, the inhibition of miR-486-5p in cells treated with etoposide resulted in a reduction of cells in G2/M (Figure 4c).

M-phase arrest induced after nocodazole treatment was used to confirm that the increase in the G1/S population is not caused by cells escaping G2/M arrest and re-entering the cell cycle. Nocodazole-treated cells had a higher proportion of cells remaining in G1 following miR-486-5p inhibition, with or without DNA damage (Supplementary Figure S2). This suggests that miR-486 contributes to G1/S cell-cycle checkpoint control.

Ankyrin proteins are composed of three parts:^34, 35^ an N-terminal membrane-binding region containing multiple ankyrin repeat domains, a central spectrin-binding region and a C-terminal regulatory region. Examination of the ankyrin-1 protein sequence revealed a death domain within the C-terminus (Supplementary Figure S3). Death domains are known to interact with components of the apoptosis pathway.^36^ We therefore examined if the induction of ankyrin-1 protein could induce apoptosis. We did not observe any cleaved caspase-3 following either induction or siRNA depletion of *ANK1* (Figure 4a). Similarly, miR-486 expression in the presence or absence of etoposide treatment did not induce caspase-3 activation despite previous work, implicating miR-486 in augmenting DDR.^25^ To investigate further, we conducted caspase-3/7 activity assays using cell lysates and annexin V-FITC staining of live cells, but failed to observe any increase in caspase activity or apoptosis (data not shown). Similarly, experiments to test if increased expression of the death domain-containing ankyrin-1 protein might change the ability of cells to engage the extrinsic apoptosis pathways^37^ did not show any effects on cell death (data not shown).

Taken together, these data suggest miR-486-5p is involved in maintaining cell-cycle arrest following DNA damage. However, increased ankyrin-1 levels do not appear to affect regulation of the cell-cycle or apoptosis in damaged cells. Owing to the marked induction of ankyrin-1 protein following DNA damage, we decided to further explore other functions of ankyrin-1 following DNA damage.

### Ankyrin-1 depletion inhibits cell migration and affects the formation of actin-rich plasma membrane protrusions following DNA damage

**Figure 5:**
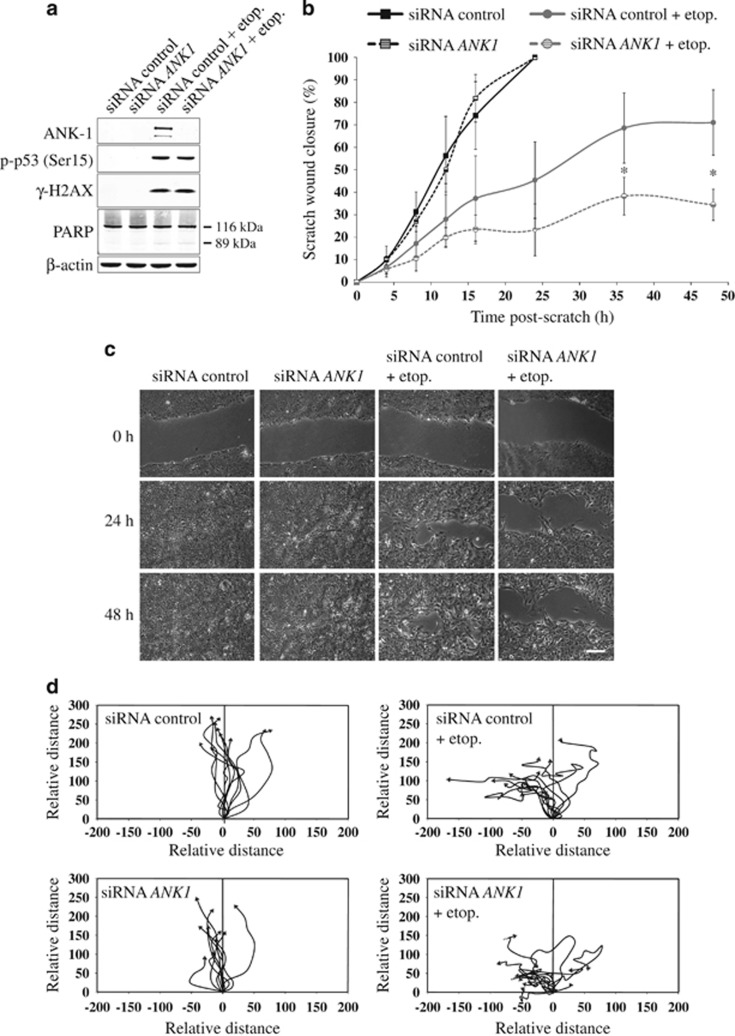
Ankyrin-1 depletion inhibits cell migration following DNA damage. (**a**) siRNA was used to deplete *ANK1*, or cells were treated with a negative control siRNA. Following siRNA treatment, DNA damage was induced with etoposide (etop.) (25 *μ*M) for 24 h. Representative western blots are shown (*n*=3). (**b**) Wound healing assays (scratch assay) was performed on confluent MCF10A cells and migration into the wound was monitored for 48 h using time-lapse microscopy. Values are mean±S.D. (*t*-test, *n*=3), *P<0.05. (**c**) Representative images of MCF10A scratch assays at the initial time of scratch (0 h) and after 24 and 48 h (*n*=3). Scale bar represents 200 *μ*m. (**d**) Representative cell tracking assays taken from 10 randomly selected cells from each condition undergoing wound healing

Upon DNA damage, cell migration is decreased^38, 39^ but is actively maintained through ATM signalling.^40^ Ankyrin-1 functions as a membrane adaptor protein, connecting cell membrane proteins to the spectrin-actin cytoskeleton, thus implicating it in cell migration.^41^ Moreover, it is well-known that p53 can inhibit cell motility through regulation of cell morphology, cell spreading and Rho signalling.^15^ Given that *ANK1* is controlled by the p53 pathway, we investigated whether *ANK1* plays a role in modulating cell motility. To this end, we performed wound-healing assays on cells treated with either control or *ANK1* siRNA, with or without etoposide (Figure 5a). Cells that had not been exposed to DNA damage underwent wound closure at the same rate in the presence or absence of *ANK1* siRNA (Figures 5b–d). As expected, cells treated with etoposide did not achieve full wound closure (Figures 5b–d). Surprisingly, cells that lacked ankyrin-1 were even more severely affected upon etoposide treatment. For example, at 36 h after wound generation, *ANK1*-expressing cells treated with etoposide achieved 68.6% scratch wound closure compared with 38.3% in the absence of ankyrin-1 (Figure 5b). Ankyrin-1 depletion also impaired cell motility in a transwell migration assay (Supplementary Figure S4). These data suggested that ankyrin-1 has a role in sustaining cell movement following DNA damage. Importantly, videos of wound healing assays (Supplementary Movies) confirmed impaired movement of *ANK1*-depleted cells following DNA damage, showing lamellipodia ruffling which is indicative of inhibited actin filament turnover.^42^ Time-lapse videos also revealed that DNA damaged cells depleted of ankyrin-1 displayed rapid extension and retraction of membrane protrusions with very little movement of the cell body. The lack of directional movement of these cells was demonstrated by cell tracking assays, which depict the movement patterns of individual cells (Figure 5d).

**Figure 6:**
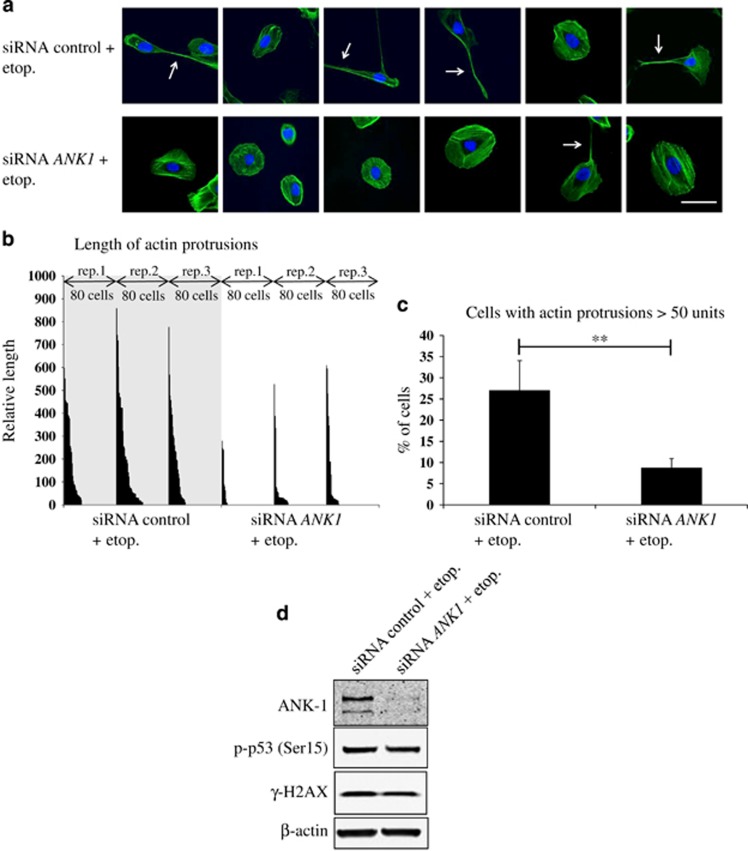
Expression of *ANK1* following DNA damage affects actin-rich plasma membrane protrusions. (**a**) Representative immunofluorescent confocal images of cells treated with either control or *ANK1*-specific siRNAs, followed by etoposide (etop.) (25 *μ*M) treatment for 24 h to induce DNA damage (*n*=3). Actin was stained using Alexa Fluor 488 phalloidin (green) and nuclei were stained using DAPI (blue). White arrows point to actin-rich plasma membrane protrusions. Scale bar represents 50 *μ*m. (**b**) Long actin processes protruding from cells (examples indicated in (**a**) with white arrows) were measured on 80 randomly selected cells from each condition. Only cells that were not in colonies were recorded. Measurements were taken from three independent repeat (rep.) experiments and actin protrusion length from the cell membrane was plotted by size (longest to shortest) for each condition in each repeat experiment, with cells lacking any protrusions being represented by a blank space (a length of zero). (**c**) Percentage of cells with actin protrusions that were greater than 50 units (arbitrary) from each condition. Values are mean±S.D. (*t*-test, *n*=3), **P<0.01. (**d**) Representative western blots (*n*=3) of proteins from cells used in confocal microscopy, showing ankyrin-1 (ANK-1) levels following DNA damage, with or without siRNA depletion

Intrigued by these observations, we proceeded to examine the cytoskeleton, specifically looking at the morphology of actin filaments in response to DNA damage in the presence or absence of ankyrin-1. Interestingly, it was observed that control cells treated with etoposide had longer, more prevalent actin-rich plasma membrane protrusions compared with etoposide-treated cells following ankyrin-1 depletion (Figure 6a). Quantification revealed that the removal of *ANK1* by siRNA decreased the proportion of cells with long actin protrusions following DNA damage from 27.1% to 8.8% (Figures 6b–d). These data suggested that ankyrin-1 may play a role in regulating actin cytoskeleton structure following DNA damage.

### Actin remodelling factors are modulated by ankyrin-1

As ankyrin-1 appeared to affect actin filament structure and cell motility, we set out to identify how upregulation of ankyrin-1 regulates actin remodelling. It is possible that increased ankyrin-1 levels alone could inhibit actin filament organisation through increased spectrin-actin binding or, alternatively, ankyrin-1 could act to modulate actin remodelling signalling pathways.

**Figure 7:**
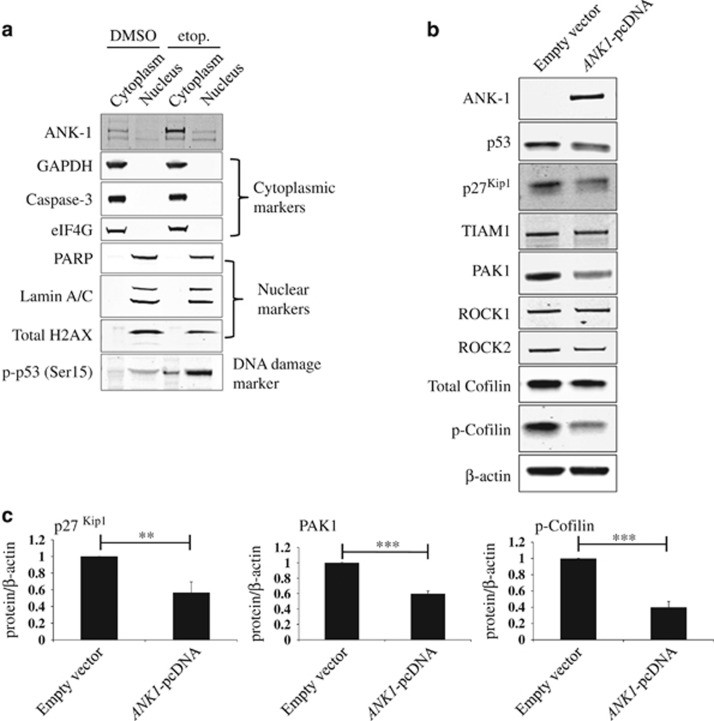
Actin remodelling factors are modulated by ankyrin-1 levels. (**a**) Western blot analysis of cells treated with control vehicle or 25 *μ*M etoposide (etop.) for 24 h. MCF10A cells were fractionated into cytoplasmic and nuclear fractions to show ankyrin-1 (ANK1) is localised to the cytoplasm following DNA damage (*n*=3). (**b**) Representative western blot analysis showing levels of actin cytoskeleton regulators following *ANK1* overexpression (*n*=3). MCF10A cells were nucleofected with an *ANK1* expression construct or with an empty vector control. Proteins involved in the actin remodelling pathway were analysed following 48 h of expression. (**c**) Western blot quantification of p27^Kip1^, p-cofilin and PAK1 for experiments shown in (**b**) following normalisation to *β*-actin levels. Values are mean±S.D. (*t*-test, *n*=3), **P<0.01 and ***P<0.001

Since ankyrin repeats (ANK) have been shown to facilitate nuclear import for some proteins,^43^ we first investigated the localisation of ankyrin-1 protein following DNA damage. We found that the ankyrin-1 protein was present predominantly in the cytoplasmic compartment (Figure 7a). We then investigated the Rho GTPase signalling pathway, which controls the remodelling of the actin cytoskeleton.^17^ To ensure the changes in this pathway were specific to increased levels of ankyrin-1 and not due to other events triggered by DNA damage, we overexpressed *ANK1* in MCF10A cells (Figure 7b). We observed a strong reduction in PAK1 levels and also a reduction in the phosphorylation of the actin-severing protein cofilin (Figures 7b and c). Both of these proteins are key regulators of actin remodelling.^17^ Examination of the tumour suppressor p27^Kip1^, which has recently been implicated in cell migration by regulating RhoA,^44^ revealed that it too was significantly reduced upon *ANK1* overexpression (Figures 7b and c).

### The impact of *ANK1* expression in cancer patients

To understand the potential clinical relevance of *ANK1* expression levels, we analysed gene expression data from cancer patients using a database tool.^45^ This analysis interrogates gene expression data and identifies correlations with patient outcome. Since the p53 pathway controls *ANK1* expression, we divided the patient cohort into two groups: the first, where a positive correlation between *TP53* and *ANK1* expression is present, and the second, where it is absent or negative. This analysis allowed us to ask if the link between *TP53* and *ANK1* was significant in a clinical setting. Importantly, we found that patients with a high positive correlation between *TP53* and *ANK1* showed increased survival (Supplementary Figure S5).

**Figure 8:**
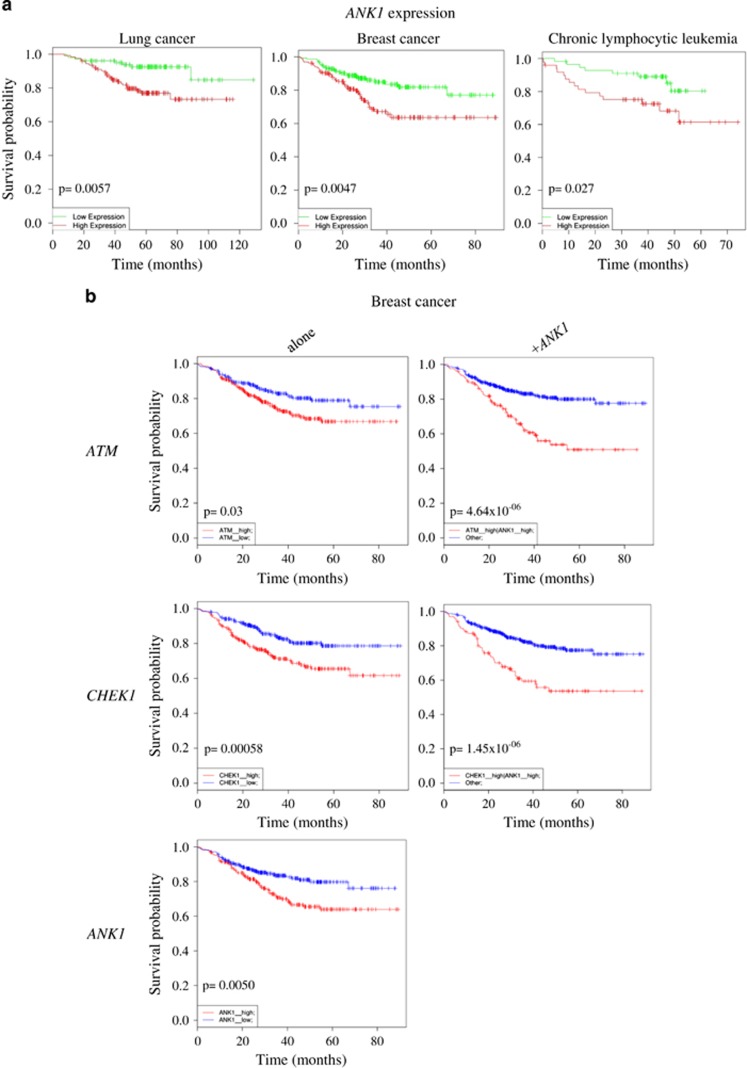
*ANK1* expression in cancer patient survival. (**a**) Kaplan–Meier survival plots of patients with lung cancer (GEO data set: GSE31210), breast cancer (GEO data set: GSE25055) and chronic lymphocytic leukaemia (GEO data set: GSE22762) with either high (red) or low (green) *ANK1* expression. Data obtained using the PPISURV database tool using different Gene Expression Omnibus (GEO) data sets. (**b**) Correlation between expression of DNA damage response regulators (*ATM* and *CHECK1*) and survival in breast cancer patients (GEO data set GSE25066), and the synergistic effect of high *ANK1* levels. High expression of genes shown in red or low in blue. Data obtained using SynTarget bioinformatics analysis tool (www.bioprofiling.de)

Having established the relationship between *TP53* and *ANK1* in the clinical setting, we next asked how *ANK1* expression alone relates to patient survival. Interestingly, we found that high *ANK1* expression correlated with decreased survival in lung cancer, breast cancer and CLL (Figure 8a). Since we have demonstrated *ANK1* plays a role in cell motility following DNA damage (Figures 5 and 6), we hypothesised that high levels of *ANK1* could accelerate the metastasis of cancer cells that are resistant to chemotherapy. To examine this we looked at the relationship between *ANK1* and genes associated with DNA repair, such as *ATM* and *CHEK1*. Indeed, we identified that high levels of both *ANK1* and either *ATM* or *CHEK1* have a negative additive effect on survival (Figure 8b). These data suggest that *ANK1* is connected with the DDR and its expression has clinical significance.

## Discussion

Previous work investigating the function of miR-486 demonstrated roles in skeletal muscle development,^32^ regulation of NF-κB signalling^46^ and the modulation of phosphoinositide-3-kinase (PI3K)/Akt signalling.^47^ In this study, we have shown that both miR-486 and *ANK1* are co-regulated by p53 and are both induced following DNA damage. Previously, miR-486 had been shown to be DDR induced^48^ and p53 regulated.^29^ However, these studies overlooked the induction of its host gene, *ANK1*. Our data show that *ANK1* is induced to a greater extent than the embedded miRNA following DNA damage. Genome-wide ChIP analysis for p53-binding sites has identified a p53-binding motif upstream of *ANK1.*^49^

MiR-486 has been shown to be controlled by the transcription factor MRTF-A at a promoter immediately upstream of exon 39a within the *ANK1* gene, which also allows for the muscle-specific production of sAnk1 (ANK1.5).^47, 50, 51^ Concurrently, miR-486 has also been shown to be regulated by a p53 promoter upstream of the *ANK1* gene in lung cancer.^29^ Our work shows that p53 controls both miR-486 and full-length *ANK1* expression following DNA damage. It is known that the *ANK1* gene is regulated by erythroid transcription factor GATA-1 and transcription factor NF-E2,^52^ but its expression in relation to p53 has never been examined. It is not unreasonable to assume that the same p53-binding site located 21.4 kb upstream of *ANK1*, previously shown to regulate miR-486,^29^ may control the expression of both transcripts. However, we cannot rule out the possible usage of other uncharacterised p53-binding sites.

Previous reports demonstrated that the overexpression of miR-486 results in inhibition of cell growth and blocks cell-cycle progression,^25, 29^ but the effect of miR-486 on cell-cycle arrest following DNA damage has not been examined. We investigated this using non-tumorigenic MCF10A cells that arrest at G2/M following DNA damage, and observed that inhibition of miR-486-5p resulted in a reduction of cells in G2/M. This suggests that, similar to the miR-34 family,^14^ miR-486-5p may play a role in the maintenance of cell-cycle arrest following DNA damage. Further investigation is required to identify mRNA targets of miR-486-5p, the repression of which would facilitate cell-cycle arrest during DDR.

Intriguingly, whereas miR-486-5p leads to the inhibition of cell-cycle progression, ankyrin-1 appears to play no role in cell-cycle maintenance, but rather has an effect on actin remodelling and facilitating cell motility. Indeed, the ankyrin protein has been implicated in cell migration.^53^ Ankyrins are highly conserved adaptor proteins that connect a variety of membrane-spanning proteins with the spectrin-actin cytoskeleton.^41^ As ankyrin-1 has been shown to bind to TIAM1, which in turn stimulates GDP/GTP exchange activity on Rac1,^53^ we looked at a panel of molecular components in the actin remodelling pathway. We found that overexpression of *ANK1* severely hampered the expression of PAK1, p27^Kip1^ and phospho-cofilin levels, but had little effect on levels of ROCK1/ROCK2 or TIAM1. It is known that cofilin, along with ARP2/3 and profilin, is one of the main effectors of actin remodelling. Therefore, we speculate that the lack of actin protrusions and perturbed migratory behaviour in DNA damaged cells lacking ankyrin-1 may be attributed to ankyrin-1 levels modulating components of the Rho GTPase signalling pathway. This ultimately results in impaired cell motility. The physiological reason for the apparent facilitation of cell migration as a result of *ANK1* upregulation following DNA damage is currently unclear; however, it is in line with other previously documented observations.^40^

In summary, we have shown how p53 can regulate a single transcript that produces two distinct molecular units with very different functions: a structural protein to facilitate motility and a co-expressed miRNA to attenuate cell-cycle progression following DNA damage. We also implicate a novel role for ankyrin-1 in the DDR. In line with our findings, a lack of *TP53*/*ANK1* correlation predicts a poor patient outcome in clinical data sets. Importantly, we found that higher *ANK1* levels alone correlated with lower tumour-free patient survival in the clinical setting. Moreover, high expression levels of *ANK1* and DNA repair genes, such as *ATM* and *CHEK1*, in combination, are additive in predicting negative outcome. In light of the role *ANK1* plays in cell motility post-DNA damage, we postulate that high levels of *ANK1* could accelerate the metastasis of cancer cells that are resistant to chemotherapy. This suggests that *ANK1* expression might potentially be used as a prognostic marker in patients with specific tumours.

## Materials and Methods

### Cell culture

MCF10A cells were cultured in DMEM/F12 (Gibco, Grand Island, NY, USA) with 5% horse serum, 1% glutamine, 20 ng/ml recombinant human EGF (Peprotech, Rocky Hill, NJ, USA), 10 *μ*g/ml insulin, 500 ng/ml hydrocortisone and 10 ng/ml cholera toxin. MCF7 and A549 cells were cultured in DMEM with 10% fetal bovine serum (FBS) and 1% l-glutamine. The p53/R175H doxycycline-inducible H1299 cells were described previously^16^ and were cultured in DMEM using 10% tetracycline-free FBS and 1% glutamine. Gene expression of p53/R175H was induced by culturing cells in 2 *μ*g/ml doxycycline for 48 h. RPE cells were cultured in DMEM/F12 with 10% FBS, 1% glutamine and 0.16% sodium bicarbonate. All cells were grown at 37 °C in a 5% CO_2_ humidified incubator.

### DNA damage and p53 induction

DNA damage was achieved using either 25 *μ*M etoposide (24 h) (Cayman, Ann Arbor, MI, USA) in dimethyl suphoxide (DMSO), 400 nM doxorubicin hydrochloride (0–48 h) (Sigma, St. Louis, MO, USA), 10 *μ*g/ml cisplatin (24 h) (Sigma) or 300 nM mitomycin C (24 h) (Sigma). IR was produced by an Xstrahl X-Ray irradiator: Xstrahl (Camberley, UK) and ultraviolet light was produced using a UV crosslinker CX-2000 (UVP, Upland, CA, USA). The p53 activator nutlin-3 (Sigma) was prepared in DMSO and used at 10 *μ*M for 24 h.

### Western blotting and antibodies

Cells were harvested in RIPA buffer (150 mM NaCl) and lysates were sonicated for 2.5 min prior to clarification. Total protein (20–40 *μ*g) was separated on polyacrylamide gels and transferred onto nitrocellulose membrane, 0.45 *μ*m. All antibodies were used at dilutions recommended by the manufacturer. Antibodies used were: ANK1 (Novus Biologicals, Littleton, CO, USA, NBP1-71805) – for detection of endogenous protein, ANK1 (Atlas Antibodies, Stockholm, Sweden, HPA056953) – for detection of cloned protein, sAnk1 (Abcam, Cambridge, UK, ab58698) – for detection of the small isoform, Total p53 (Santa Cruz, Dallas, TX, USA, sc-126), Phospho-p53 (Ser15) (Cell Signalling, Danvers, MA, USA, #9284), MDM2 (Santa Cruz, sc-965), Total H2A.X (Cell Signalling, #2595), Phospho-histone (*γ*) H2A.X (Ser139) (Cell Signalling, #9718), PARP (Cell Signalling, #9542), B-actin (Sigma, A5441), p21^Waf1/Cip1^ (Santa Cruz, sc-397), Phospho-ATM (Ser1981) (Abcam, ab81292), Caspase-3 (Cell Signalling, #9662), GAPDH (Santa Cruz, sc-32233), eIF4G (Cell Signalling, #2498), Lamin A/C (Santa Cruz, sc-7292), p27^Kip1^ (Cell Signalling, #3686), PAK1 (Bethyl Laboratories, Montgomery, TX, USA, A301-259AT), Total cofilin (Cell Signalling, #5175), Phospho-cofilin (Ser3) (Cell Signalling, #3313), ROCK1 (Cell Signalling, #4035), ROCK2 (Bethyl Laboratories, A300-047A-T), TIAM1 (Bethyl Laboratories, A300-099AT) and Vinculin (Abcam, ab18058). IR-Dye-labelled secondary antibodies (LI-COR) were used to detect primary antibodies. Blots were scanned with a LI-COR laser-based image detector: LI-COR Biosciences, (Lincoln, NE, USA) and analysed using Image Studio software (v.2.1).

### Small RNA library preparation

Total RNA was purified using Trizol reagent (Invitrogen, Carlsbad, CA, USA). Small RNA libraries were prepared using the TruSeq Small RNA kit (Illumina, San Diego, CA, USA) with 0.5 *μ*g total RNA. Libraries were sent to The Genome Analysis Centre (TGAC), Norwich. The small RNA libraries were checked on a BioAnalyser (Agilent DNA 2100 chip, Agilent, Santa Clara, CA, USA) and LabChip GX, followed by quantification. Libraries were pooled and sequenced on the Illumina HiSeq 2500 (Rapid-Run mode, cBot clustering) platform using 50 bp single end reads.

### Bioinformatics analysis of deep sequencing data and accession numbers

All sequencing files were transformed from FASTQ to FASTA format and the adaptors were removed by matching the first 8 nt of adaptor sequence (TGGAATTC). These reads were then then mapped to mature human miRNA sequences from miRBase version 20^54^ using PatMaN^55^ to obtain counts for all annotated miRNAs in each of the samples. Differentially expressed miRNAs were called using the edgeR package.^56^ A *P*-value of 0.05 was used as a cutoff to analyse microRNAs that were upregulated following DNA damage. Deep sequencing data are available from National Center for Biotechnology Information (NCBI) Sequence Read Archive (SRA) accession SRP064590.

### Quantitative PCR and TaqMan assays

MicroRNAs were quantified using TaqMan assays (Applied Biosystems, Foster City, CA, USA) with 100 ng input total RNA. MicroRNA and sRNA-specific reverse transcription primer and probe reagents used were: hsa-miR-486-5p (#001278), hsa-miR-34a-5p (#000426), hsa-miR-34c-5p (#000428) and U6 snRNA (#001973). SYBR green (Applied Biosystems) based quantitative PCR (qPCR) was used for mRNA quantification. Superscript III reverse transcriptase (Invitrogen) was used to convert 1 *μ*g total RNA to cDNA using random primers. cDNA was used as a template in qPCR with gene-specific primers: *ANK1*: forward 5′-TGAAGACGGGAGCCTCGAT-3′ and reverse 5′-GGGGTCTCCACTTTCACGTT-3′ *p21*^Waf1/Cip1:^ forward 5′-GGAGACTCTCAGGGTCGAAA-3′ and reverse 5′-GGATTAGGGCTTCCTCTTGG-3′ and GAPDH: forward 5′-AGCCACATCGCTCAGACAC-3′ and reverse 5′-GCCCAATACGACCAAATCC-3′.

The ΔΔCT method was used to calculate fold-change between the control sample and the other conditions using U6 snRNA as an internal control gene for microRNAs and GAPDH for mRNAs.

### Statistical analysis

Where stated, statistical significance was analysed using the Student's *t*-test (two sample, two tailed, unpaired) and expressed as a *P*-value. In all bar charts and graphs, error bars represent the standard deviation of relative values/fold-changes from three experiments. Statistical significance for cancer patient data analysis was calculated using the relevant bioinformatics tool (see below).

### siRNAs and transfection

SiRNA transfections were performed using Lipofectamine RNAiMAX (Invitrogen) and Opti-MEM+GlutaMAX (Gibco). The negative control siRNA used in all experiments was the ON-TARGETplus non-targeting siRNA #3 (Thermo Scientific, Waltham, MA, USA, D-001810-03-50), (+)5′-UCAGAAAACAUGUAAACCA(dTdT)-3′, (−)5′-UGGUUUACAUGUUUUCUGA-3′. The p53 targeting siRNA was custom ordered from Eurofins (Luxembourg City, Luxembourg), (+)5′-GACUCCAGUGGUAAUCUAC(dTdT)-3′, (−)5′-GUAGAUUACCACUGGAGUC(dTdT)-3′, and transfected at 50 nM twice over 2 days. The *ANK1* targeting siRNA was a Silencer Select siRNA (Ambion, s1364), (+)5′-AAAUCAGUUUAACACCUUA(dTdT)-3′, (−)5′-UAAGGUGUUAAACUGAUUU(dCdA)-3′ and was transfected once at 20 nM.

### FACS

For cell-cycle analysis, cells were fixed in ice cold 70% ethanol/30% PBS and left at −20 °C for 6–12 h. Cells were then washed in PBS and stained with 10 *μ*g/ml propidium iodide and 0.1 mg/ml RNase A in PBS and left at 37 °C for 30 min, followed by overnight incubation at 4 °C. Propidium iodide incorporation into DNA was measured using the BD FACSCanto II (BD Biosciences, San Jose, CA, USA) with the 488 nm laser, measuring forward scatter, side scatter and red fluorescence. 10 000 cells were measured for each experimental condition.

### Wound healing assay (scratch assay)

MCF10A cells were transfected with the appropriate siRNA and then treated the following day with either DMSO or 25 *μ*M etoposide for 24 h. Cells were allowed to grow to full confluency in six-well plates. A linear scratch was made using a 10 *μ*l pipette tip and cell migration into the wound was monitored for 48 h using a phase contrast time-lapse microscope at × 5 magnification. Images were taken at three different points along the scratch at 10 min intervals using MetaMorph Microscopy Automation & Image Analysis Software (v.7.8): Molecular Devices (Sunnyvale, CA, USA). Scratch wound closure was measured at specific time points along each scratch using the ImageJ software: National Institutes of Health, (Bethesda, MD, USA). Cell tracking was achieved using the Manual Tracking plugin on ImageJ.

### Immunofluorescence confocal microscopy

Cells were grown on coverslips at low confluency and transfected with the appropriate siRNA. The following day cells were treated with either DMSO or 25 *μ*M etoposide for 24 h. Cells were then fixed using 4% formaldehyde in PBS for 10 min and permbealised with 0.25% Triton X-100 in PBS for a further 10 min. Cells were blocked in 5% goat serum and 0.1% Triton X-100 in PBS for 1 h, followed by staining with Alexa Fluor 488 phalloidin (Life Technologies, Carlsbad, CA, USA) according to the manufacturer's instructions. Coverslips were mounted using Vectashield hard set mounting medium with DAPI (Vector Laboratories, Burlingame, CA, USA). Confocal microscopy was performed using a Zeiss LSM 510 microscope at × 63 magnification, equipped with ZEN software (Zeiss, Oberkochen, Germany). Only isolated cells (not in colonies) had their protruding actin processes measured using ImageJ software.

### Cytoplasmic and nuclear fractionation

Cells were washed twice in PBS and pelleted by centrifugation at 250 × *g* for 2 min, then resuspended in fractionation buffer (10 mM HEPES pH 7.9, 10 mM KCl, 1.5 mM MgCl_2_, 0.34 M sucrose, 10% glycerol, 1 mM DTT, 1 × protease inhibitor (Roche, Basel, Switzerland), 10 mM *β*-glycerophosphate, 50 mM NaF, 15 *μ*M MG-132), followed by addition of 0.1% Triton X-100. Cells were incubated on ice for 8 min followed by centrifugation at 1300 × *g*, 4 °C for 5 min, followed by collection and clarification of the supernatant (cytoplasmic) fraction. The pellet (nucleus) was washed twice with fractionation buffer and lysed in RIPA buffer (same volume as cytoplasmic fraction), followed by sonication for 5 min and clarification. Equal volumes of nuclear and cytoplasmic lysate were analysed via western blot.

### *ANK1* cloning and nucleofection

The *ANK1* gene was cloned from ORFeome Collaboration vector BC156401 (Thermo) into pcDNA3.1(+) (Invitrogen) using primers: Forward: 5′-CGCGGAATTCATGCCCTATTCTGTGGGCTTC-3′ (with *Eco*RI restriction site) and Reverse: 5′- CGGCCTCGAGTCACTTGTCGTCATCGTCTTTGTAGTCGGGGTTGGGTGTCGAGGTG-3′ (with *Xho*I site and FLAG tag). PCR was performed using Phusion DNA Polymerase (NEB) in HF buffer with DMSO. Temperature cycling parameters were: 98 °C for 1 min, followed by four cycles of: 98 °C – 10 s, 50 °C – 20 s, 72 °C – 5 m 30 s, followed by 25 cycles of: 98 °C – 10 s, 60 °C – 20 s, 72 °C – 5 m 30 s, followed by a final 10 min extension at 72 °C. The entire cloned *ANK1* sequence was checked by Sanger sequencing following cloning. The pcDNA-*ANK1*-FLAG construct was nucleofected using programme T-020 (AMAXA nucleofector kit L) (Lonza, Basel, Switzerland) into 1 million MCF10A cells using 1.5 *μ*g plasmid. Cells were harvested 48 h after nucleofection.

### Datamining of gene expression data

The analyses of translational relevance of *ANK1* have been performed with the BioProfiling.de online datamining tools^45^ using publically available clinical gene expression data from the GEO omnibus repository. The PPISURV^57^ tool was used to correlate gene expression data with cancer patient survival. To demonstrate translational relevance of potential association between *ANK1* and DNA damage genes we used the SynTarget tool. SynTarget estimates the synergy of gene expression of two genes on survival of cancer patients. Patients in the clinical data sets are split into four groups with respect to the expression of the *ANK1* and CHK1/ATM genes (high–high, high–low, low–high and low–low). Next, each group is tested *versus* other samples to find any statistical differences in survival outcome (i.e. high–high *versus* others, high–low *versus* others and so on).

### Determining p53/*ANK1* correlation and survival outcome in cancer patients

In contrast to the standard approach, where patients are grouped based on high/low expression of a gene, in this case, patients are grouped based on presence/absence of correlation between p53/*ANK1* genes. Namely, in the first group we selected patients so as to maximise positive correlation of p53/*ANK1*, while all the other patients formed the second group. To identify statistical differences in survival outcome between the two groups of patients, the R statistical package was used to perform statistical tests and to derive the *P*-value.

## Abbreviations

ANK1: ankyrin-1
ATM: ataxia telangiectasia mutated
CHEK1: checkpoint kinase 1
CLL: chronic lymphocytic leukaemia
DDR: DNA damage response pathway
DMSO: dimethyl sulphoxide
Dox.: doxorubicin
DSB: DNA double-strand break
Etop.: etoposide
G1/G2: gap phases of the cell-cycle
*γ*-H2A.X: phosphorylated H2A histone family member X
IR: ionising radiation
miRNA: microRNA
MDM2: mouse double minute 2 homologue
p53: tumour suppressor protein p53
PAK1: p21 protein (Cdc42/Rac)-activated kinase 1
PARP: poly (ADP-ribose) polymerase
RPE: retinal pigment epithelium cells
S-phase: synthesis phase of the cell cycle
sAnk1/ANK1.5: shorter muscle-enriched isoform of ankyrin-1
siRNA: small interfering RNA
TIAM1: T-lymphoma invasion and metastasis 1
UVC: ultraviolet-C irradiation
