# Supplementary material for: The cytoskeleton adaptor protein ankyrin-1 is upregulated by p53 following DNA damage and alters cell migration
Source: Cell Death Dis. 2016 Apr 7;7(4):e2184–. doi: 10.1038/cddis.2016.91 (PMC4855670; doi:10.1038/cddis.2016.91)
Supplement: Supplementary Information [file cddis201691x1.docx]

**Supplementary Figure Legends**

**Supplementary Figure S1.**

Western analysis of cells treated with DNA damaging agents (etoposide or doxorubicin) or the apoptosis inducer TRAIL. Cleaved PARP and cleaved caspase-3 fragments are indicative of apoptosis activation in cells. The lack of PARP cleavage in etoposide and doxorubicin treated cells demonstrates that DDR activation is not inducing apoptosis.

**Supplementary Figure S2.**

(A) miR-486-5p contributes to G1/S cell-cycle checkpoint control. MCF10A cells were transfected with a negative control 2′-O-methyl inhibitor or a miR-486-5p 2′-O-methyl inhibitor. After 24h, G2 cell cycle arrest was induced using 0.1µg/ml nocodazole. Cell-cycle analysis was performed by flow cytometry with propidium iodide staining (n=3). (B) DNA damage was induced by 25µM etoposide treatment of control 2′-O-methyl or miR-486-5p 2′-O-methyl treated cells, with or without nocodazole treatment, followed by FACS (propidium iodide) analysis (n=3). (C) Representative Western blots of proteins from cells used for FACS analysis. For all bar charts, values are mean +/- SD (t-test, n=3), p<0.05 (*), p<0.01(**) and p<0.001 (***); n.s. not significant.

**Supplementary Figure S3.**

(A) A death domain is present in the ankyrin-1 protein. Protein sequence annotation ankyrin-1 shows that a death domain is present in the C-terminal regulatory domain. Sequence annotation done using Simple Modular Architecture Research Tool (SMART) (http://smart.embl-heidelberg.de/) using ankyrin-1 isoform 1 [Homo sapiens] (NCBI Reference Sequence: NP_065209.2). (B) Ankyrin-1 death domain sequence alignment showing amino acid sequence conservation between different species. Alignment done using Clustal Omega (EMBL-EBI) with ankyrin-1 orthologs from Homo sapiens (NP_065209.2), Mus musculus (NP_001104253.1), Xenopus tropicalis (XP_012814593.1) and Danio rerio (XP_009300281.1). (C) Cladogram made using molecular data shown in B.

**Supplementary Figure S4.**

(A) Real-time, label-free monitoring of cellular migration using the xCELLigence RTCA System. MCF10A cells were treated with either control or *ANK1* specific siRNAs. Following siRNA treatment, DNA damage was induced with etoposide (etop.) (25µM) for 24h, while control cells were treated with DMSO. Cell migration was monitored in quadruplicate for each condition over 48h and measurements were taken every 10min. Error bars are SE, n=3. (B) Cell index (electrical-impedance) is shown for etoposide treated cells, with or without ankyrin-1 depletion at three time-points. Values are mean +/- SE (t-test, n=3), p<0.05 (*), p<0.01(**) and p<0.001 (***); n.s. not significant. (C) Staining of migratory cells (blue) treated under the same conditions as above, plated in transwell (Boyden) chambers in a separate experimental assay to show level of migration at 24h. (D) Representative Western analysis from cells used in xCELLigence and transwell migration staining assays.

**Supplementary Figure S5.**

Positive p53/*ANK1* correlation gives good survival in cancer patients. (A-H) Different cancer data sets analysed for correlation of p53 and *ANK1* expression profiles: cohorts 1 show positive correlation (left panels) and cohorts 2 show negative/no correlation (middle panels). Right panels show patient survival for positive correlation groups compared with negative/no correlation groups. Gene expression analysis done on data obtained from the GEO omnibus repository.
