# Supplementary material for: The cytoskeleton adaptor protein ankyrin-1 is upregulated by p53 following DNA damage and alters cell migration
Source: Cell Death Dis. 2016 Apr 7;7(4):e2184–. doi: 10.1038/cddis.2016.91 (PMC4855670; doi:10.1038/cddis.2016.91)
Supplement: Supplementary Methods [file cddis201691x2.docx]

**Supplementary Methods**

**Nocodazole treatment**

Nocodazole (Cayman) was used at 0.1µg/ml for 24h in order to induce G2 cell cycle arrest. Cells were treated with nocodazole the day after siRNA treatment, either alone or in conjunction with 25µM etoposide. Cell-cycle analysis was achieved using propidium iodide-FACS as already described.

**Cell migration assays**

For real-time, label-free monitoring of cellular migration: migration was measured using the xCELLigence RTCA System (Acea Biosciences) according to the manufacturer’s instructions, which analyses migration using electronically integrated Boyden chambers. Following siRNA and etoposide treatment, 40 000 cells were seeded from each condition in separate wells of a CIM-Plate 16 in serum-free, EGF-free MCF10A media. Each condition was analysed in quadruplicate (4 wells per condition) for each of the three independent experiments. The lower chamber of each well was filled with normal MCF10A media containing EGF and serum, acting as a chemoattractant. Electrical impedance changes (recorded as cell index) were measured at 10min intervals for 48h. For staining of migratory cells, 25 000 cells were seeded in the upper chamber of an insert (8µM pore size, Costar) of a polycarbonate membrane transwell plate under the same experimental conditions (stated above). After 24h, cells remaining on the upper membrane were removed with cotton wool. Cells which had migrated to the lower membrane were fixed and stained using Romanowsky staining solutions (TCS Biosciences Ltd). Stained cells were visualised using an Axiovert 200M inverted microscope (Zeiss).
