# Supplementary material for: The cytoskeleton adaptor protein ankyrin-1 is upregulated by p53 following DNA damage and alters cell migration
Source: Cell Death Dis. 2016 Apr 7;7(4):e2184–. doi: 10.1038/cddis.2016.91 (PMC4855670; doi:10.1038/cddis.2016.91)
Supplement: Supplementary Figure S1 [file cddis201691x3.ppt]

## Slide 1
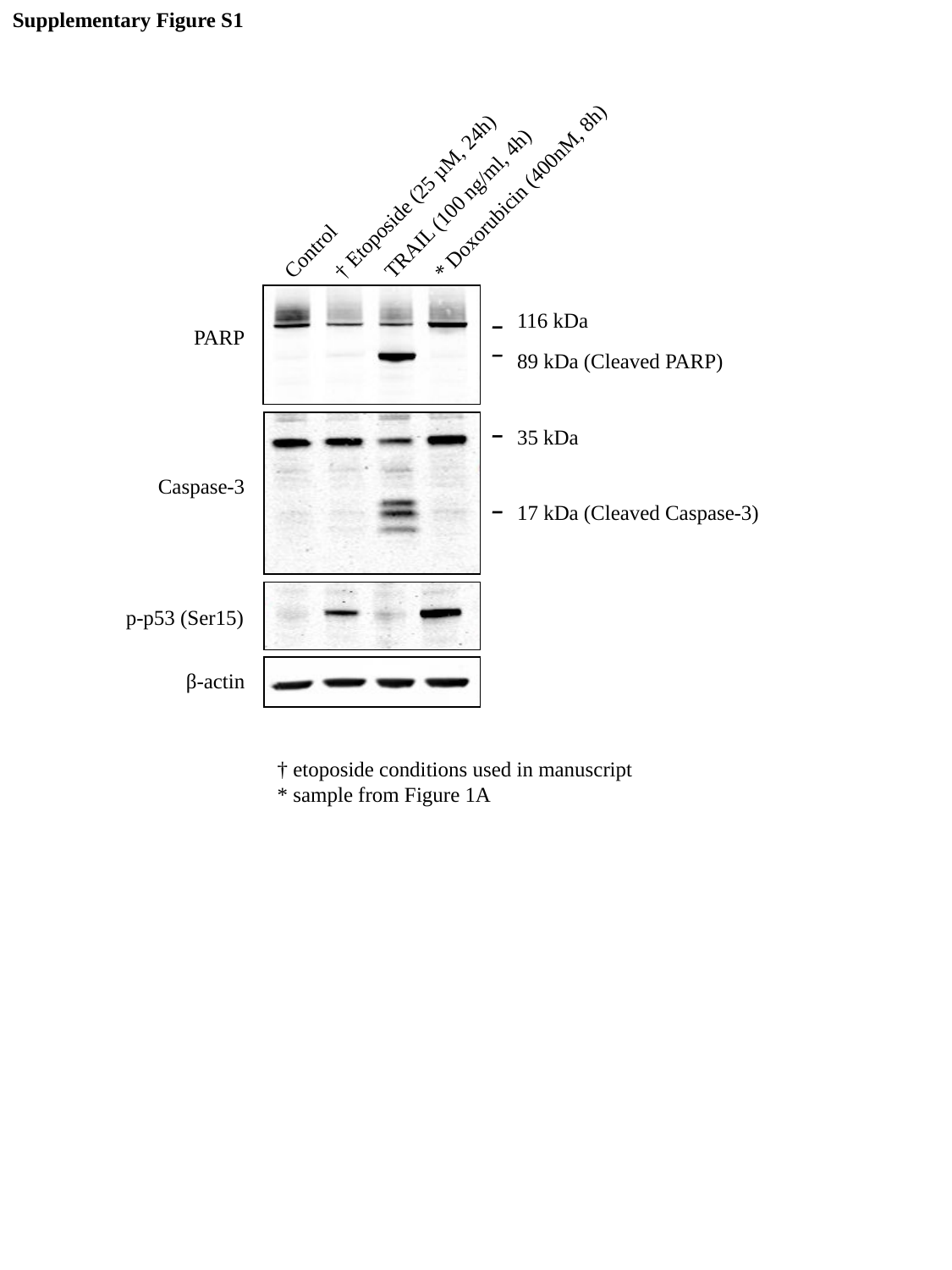

Supplementary Figure S1
† Etoposide (25 µM, 24h)
* Doxorubicin (400nM, 8h)
TRAIL (100 ng/ml, 4h)
Control
116 kDa
PARP
89 kDa (Cleaved PARP)
35 kDa
Caspase-3
17 kDa (Cleaved Caspase-3)
p-p53 (Ser15)
β-actin
† etoposide conditions used in manuscript
* sample from Figure 1A
