# Supplementary material for: The cytoskeleton adaptor protein ankyrin-1 is upregulated by p53 following DNA damage and alters cell migration
Source: Cell Death Dis. 2016 Apr 7;7(4):e2184–. doi: 10.1038/cddis.2016.91 (PMC4855670; doi:10.1038/cddis.2016.91)
Supplement: Supplementary Figure S2 [file cddis201691x4.ppt]

## Slide 1
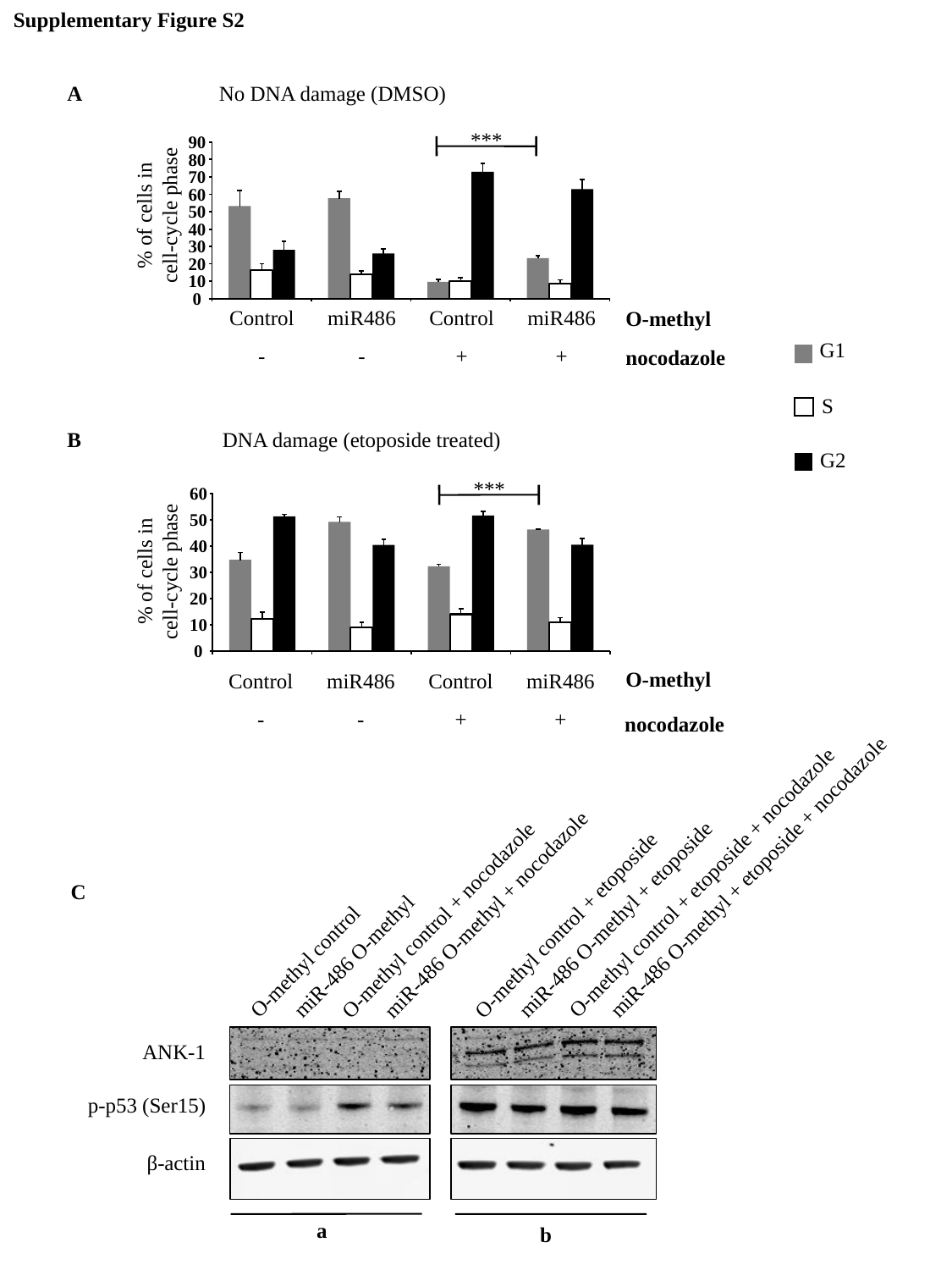

Supplementary Figure S2
A
No DNA damage (DMSO)
 ***
90
80
70
60
% of cells in cell-cycle phase
50
40
30
20
10
0
O-methyl
| Control | miR486 | Control | miR486 |
| --- | --- | --- | --- |
| - | - | + | + |
G1
S
G2
nocodazole
B
DNA damage (etoposide treated)
 ***
60
50
40
% of cells in cell-cycle phase
30
20
10
0
O-methyl
| Control | miR486 | Control | miR486 |
| --- | --- | --- | --- |
| - | - | + | + |
nocodazole
O-methyl control + etoposide + nocodazole
miR-486 O-methyl + etoposide + nocodazole
C
miR-486 O-methyl + nocodazole
O-methyl control + nocodazole
miR-486 O-methyl + etoposide
O-methyl control + etoposide
O-methyl control
miR-486 O-methyl
ANK-1
p-p53 (Ser15)
β-actin
a
b
