# Supplementary material for: The cytoskeleton adaptor protein ankyrin-1 is upregulated by p53 following DNA damage and alters cell migration
Source: Cell Death Dis. 2016 Apr 7;7(4):e2184–. doi: 10.1038/cddis.2016.91 (PMC4855670; doi:10.1038/cddis.2016.91)
Supplement: Supplementary Figure S3 [file cddis201691x5.ppt]

## Slide 1
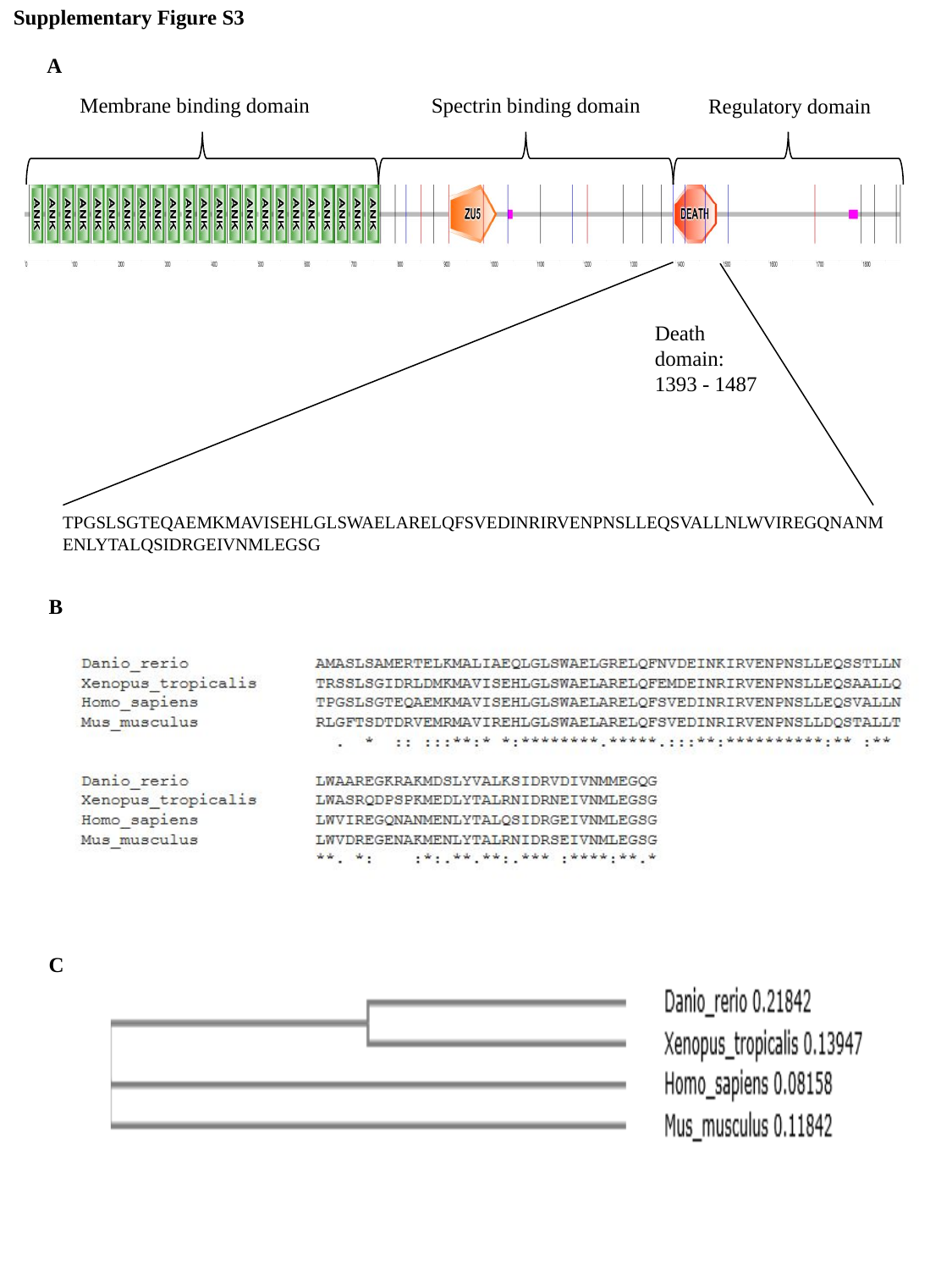

Supplementary Figure S3
A
Membrane binding domain
Spectrin binding domain
Regulatory domain
Death domain: 1393 - 1487
TPGSLSGTEQAEMKMAVISEHLGLSWAELARELQFSVEDINRIRVENPNSLLEQSVALLNLWVIREGQNANMENLYTALQSIDRGEIVNMLEGSG
B
C
