# Supplementary material for: The cytoskeleton adaptor protein ankyrin-1 is upregulated by p53 following DNA damage and alters cell migration
Source: Cell Death Dis. 2016 Apr 7;7(4):e2184–. doi: 10.1038/cddis.2016.91 (PMC4855670; doi:10.1038/cddis.2016.91)
Supplement: Supplementary Figure S4 [file cddis201691x6.ppt]

## Slide 1
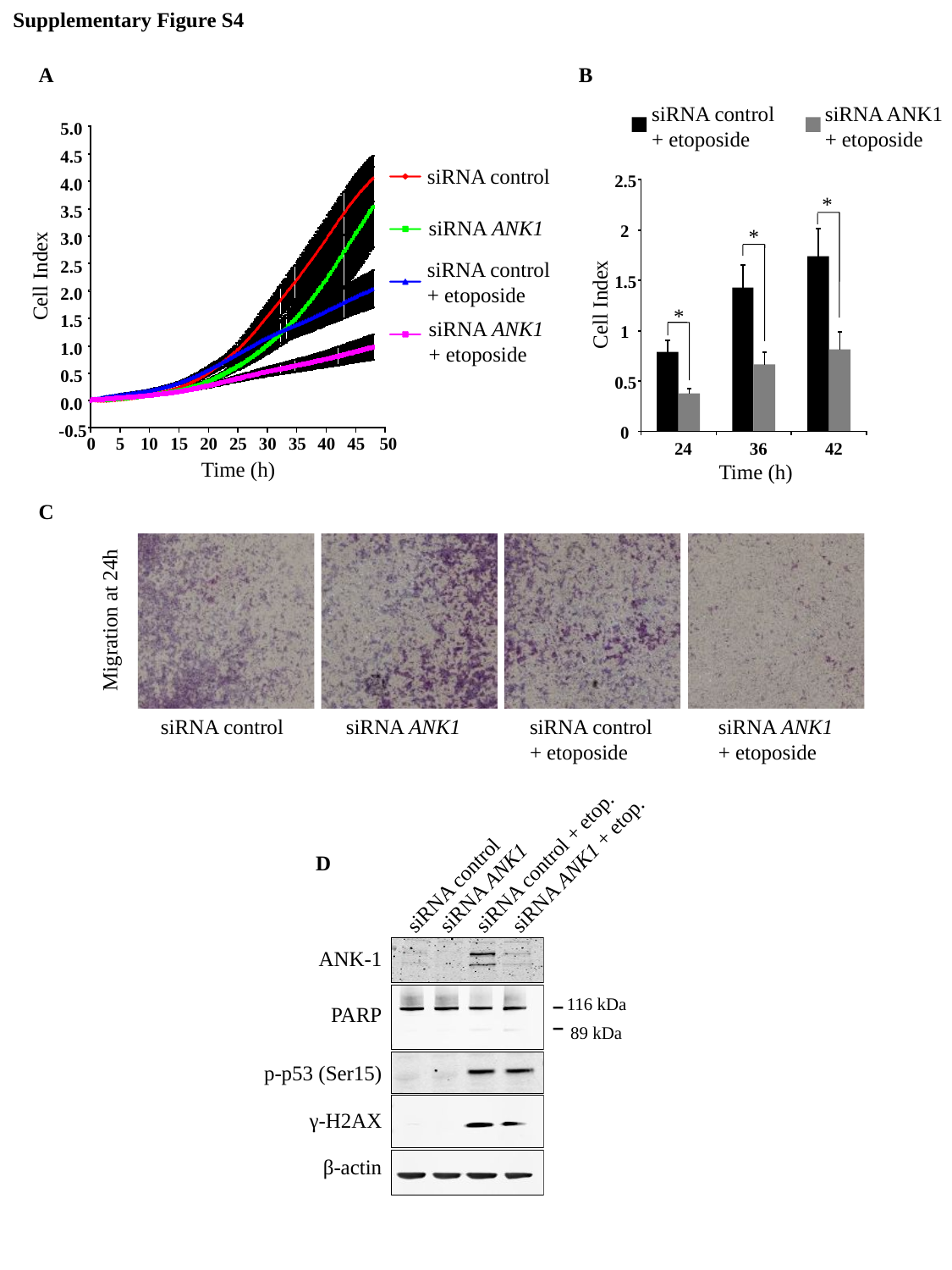

Supplementary Figure S4
A
B
siRNA control
+ etoposide
siRNA ANK1
+ etoposide
5.0
4.5
siRNA control
2.5
4.0
 *
3.5
siRNA ANK1
 *
2
3.0
2.5
siRNA control
+ etoposide
Cell Index
1.5
2.0
Cell Index
 *
1.5
siRNA ANK1
+ etoposide
1
1.0
0.5
0.5
0.0
-0.5
0
0
5
10
15
20
25
30
35
40
45
50
24
36
42
Time (h)
Time (h)
C
Migration at 24h
siRNA control
siRNA ANK1
siRNA control
+ etoposide
siRNA ANK1
+ etoposide
siRNA control + etop.
siRNA ANK1 + etop.
D
siRNA ANK1
siRNA control
ANK-1
116 kDa
PARP
89 kDa
p-p53 (Ser15)
γ-H2AX
β-actin
