# Supplementary material for: The cytoskeleton adaptor protein ankyrin-1 is upregulated by p53 following DNA damage and alters cell migration
Source: Cell Death Dis. 2016 Apr 7;7(4):e2184–. doi: 10.1038/cddis.2016.91 (PMC4855670; doi:10.1038/cddis.2016.91)
Supplement: Supplementary Figure S5 [file cddis201691x7.ppt]

## Slide 1
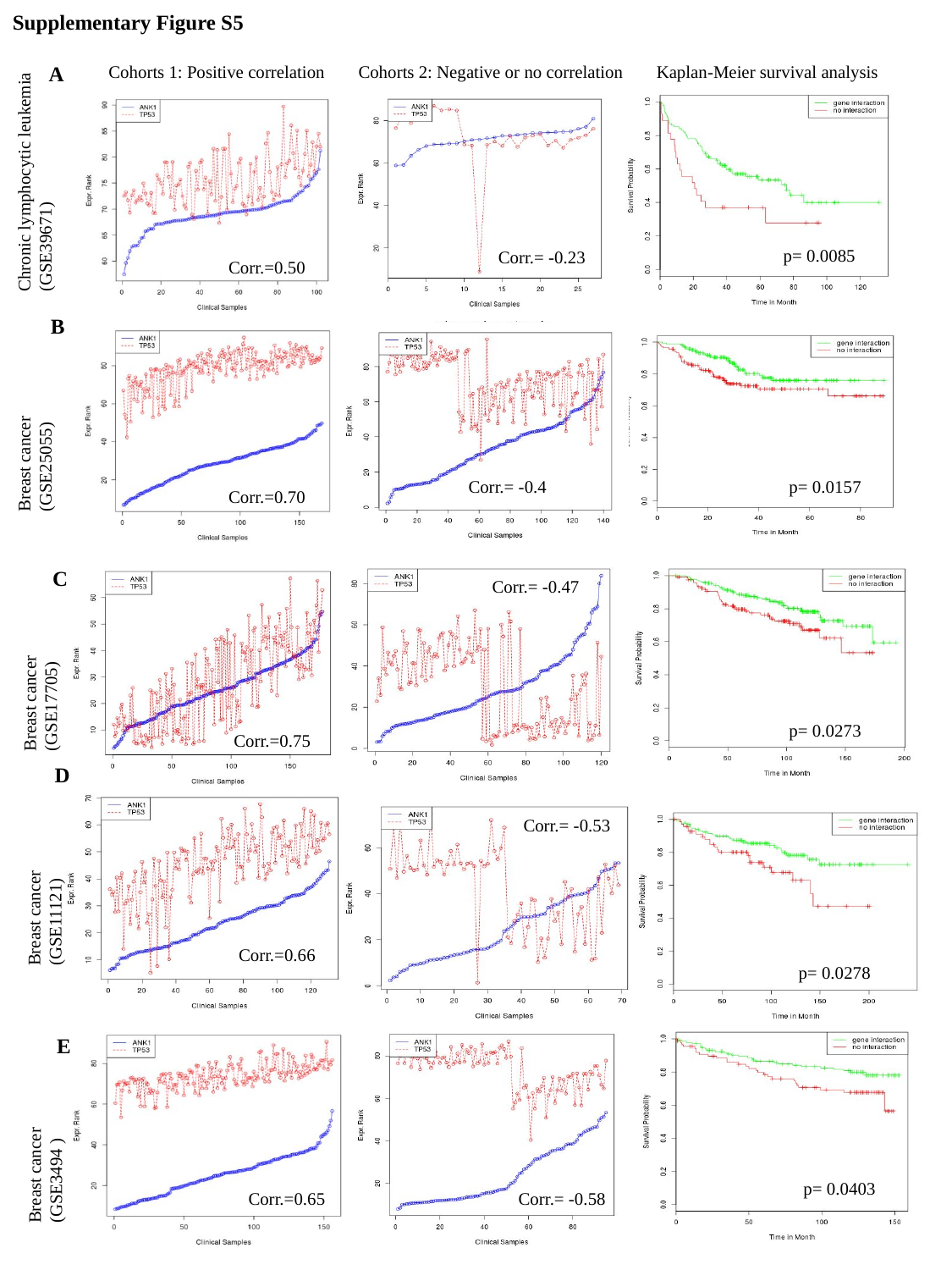

Supplementary Figure S5
A
Cohorts 1: Positive correlation
Cohorts 2: Negative or no correlation
Kaplan-Meier survival analysis
Chronic lymphocytic leukemia
(GSE39671)
p= 0.0085
Corr.= -0.23
Corr.=0.50
B
Breast cancer
(GSE25055)
Corr.= -0.4
p= 0.0157
Corr.=0.70
C
Corr.= -0.47
Breast cancer
(GSE17705)
p= 0.0273
Corr.=0.75
D
Corr.= -0.53
Breast cancer
(GSE11121)
Corr.=0.66
p= 0.0278
E
Breast cancer
(GSE3494 )
p= 0.0403
Corr.=0.65
Corr.= -0.58

## Slide 2
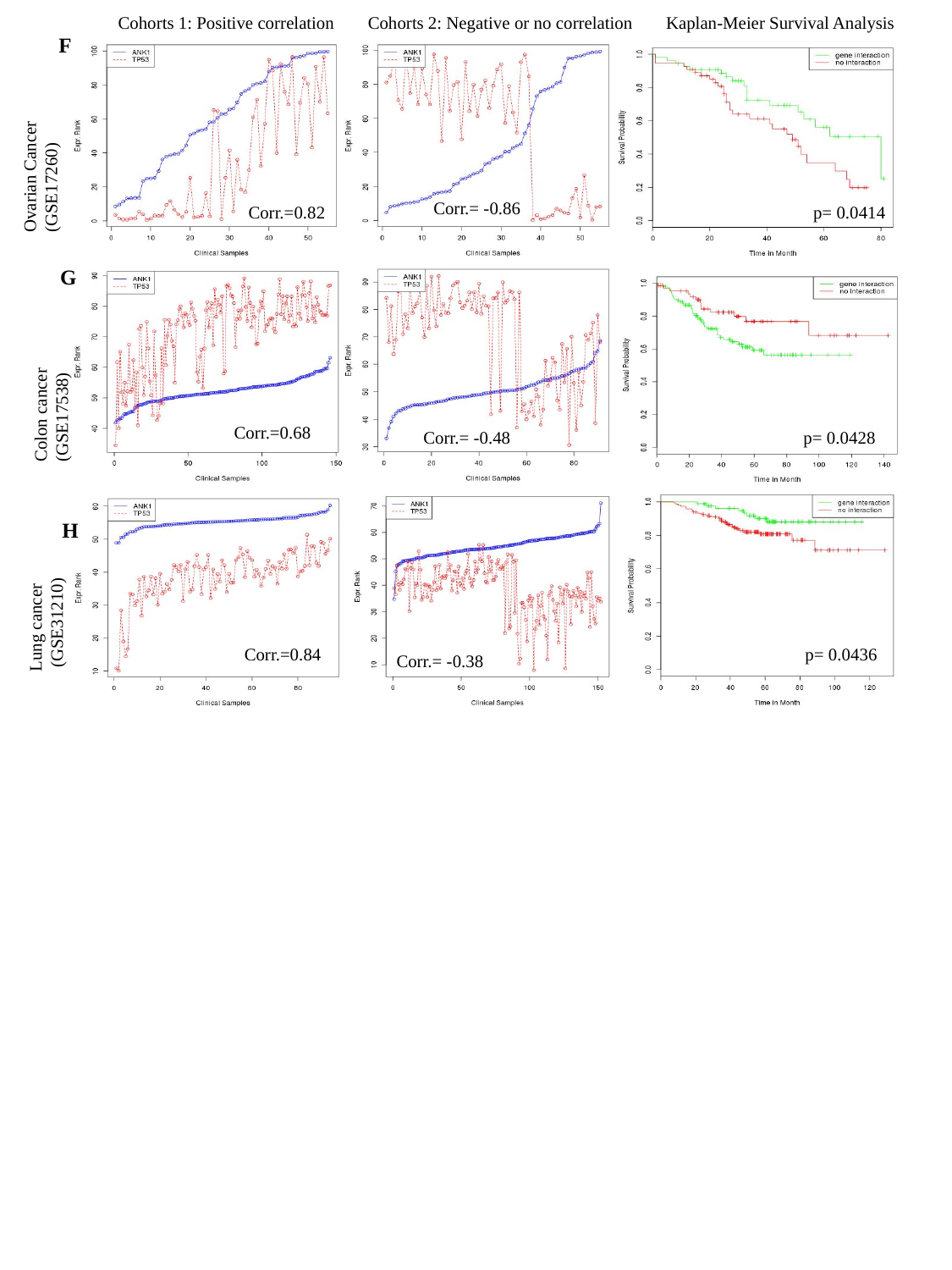

Cohorts 1: Positive correlation
Cohorts 2: Negative or no correlation
Kaplan-Meier Survival Analysis
F
Ovarian Cancer
(GSE17260)
Corr.= -0.86
Corr.=0.82
p= 0.0414
G
Colon cancer
(GSE17538)
Corr.=0.68
Corr.= -0.48
p= 0.0428
H
Lung cancer
 (GSE31210)
Corr.=0.84
p= 0.0436
Corr.= -0.38
